# Drawing conclusions: blood culture yield across clinical scenarios in hematologic malignancy

**DOI:** 10.1017/ash.2026.10784

**Published:** 2026-07-17

**Authors:** Jessica Seidelman, Patrick Tam, Julia Messina, Alicia Gray, JoAnn Liu, Kimberiey Milliam, Jonathan Huggins, Jennifer Saullo

**Affiliations:** 1 Infectious Diseases, Duke University Hospital, Durham, USA; 2 Duke University, USA; 3 Duke University School of Medicine, USA; 4 Duke University Hospital, USA

## Abstract

Patients with hematologic malignancies are excluded from most blood culture stewardship algorithms. We evaluated blood culture collection patterns and bloodstream infection incidence across clinical scenarios in this population. Blood culture yield varied substantially by indication, identifying high-yield scenarios and opportunities to refine diagnostic stewardship while preserving patient safety.

## Introduction

Bloodstream infections (BSIs) remain a leading cause of morbidity and mortality among patients with hematologic malignancies, driven by prolonged neutropenia, mucosal barrier injury, central venous access, and frequent healthcare exposures.^1,2^ Despite advances in antimicrobial prophylaxis, rapid diagnostics, and supportive care, BSI incidence in this population remains high, and early detection remains a priority.

Blood culture (BCx) diagnostic stewardship aims to optimize test utilization by balancing diagnostic yield that influences clinical decision-making against downstream harms, including excessive blood sampling, unnecessary antibiotic exposure, and excess resource use. Existing BCx stewardship algorithms have demonstrated success in immunocompetent and general hospitalized populations; however, patients with hematologic malignancies are routinely excluded from these frameworks due to lack of validation of such algorithms and perceived elevated risk of BSI-related morbidity and mortality in this population. As a result, BCxs are often obtained reflexively, including on repeated days for persistent fever, without robust data to support this practice.^3^

Few studies have examined BCx yield stratified by specific clinical scenarios in patients with hematologic malignancies. Understanding scenario-specific yield is essential to developing evidence-based stewardship approaches tailored to this high-risk population. The objective of this study was to determine the incidence of positive BCxs across common clinical indications in patients with hematologic malignancies in both inpatient and outpatient settings.

## Methods

We performed a single-center retrospective chart review of all consecutive BCxs obtained from patients with hematologic malignancies in inpatient and outpatient oncology units at Duke University Medical Center from 10/1/23–2/29/24.

All BCxs collected in adult patients (≥18 years old) with a diagnosis of hematological malignancy and/or recipient of a hematopoietic stem cell transplant during the study period were included. BCx were classified as negative, true positive, or contaminant based on standardized CDC definitions. Additional clinical data, including organism identification, number of positive culture sets, and clinical context triggering blood sampling, were also collected. BCxs were classified according to clinical indications for collection, including neutropenic fever (*T*_max_ ≥ 38.0°C), non-neutropenic fever, chills or rigors without fever, documentation of BSI clearance, and evaluation for dissemination of focal infections such as skin and soft tissue, bone, or intra-abdominal infections.

Patient demographics, underlying malignancy, and BCx characteristics were extracted from the electronic health record. The primary outcome was the incidence of true-positive BCxs by clinical scenario. We defined a BSI case as a recognized pathogen cultured from one or more BCxs.

This study was reviewed and approved by the Duke University Institutional Review Board with a waiver of informed consent.

## Results

During the study period, 1,511 BCxs were collected from 276 unique patients, with 90.5% obtained in the inpatient setting. Of these, 7.3% were adjudicated as true positives and 1.8% as contaminants.

BCxs were most collected among patients with acute myeloid leukemia (34.3%, 518/1511), multiple myeloma/plasma cell leukemia (17.3, 262/1511) and non-Hodgkin lymphoma (17.1% 259/1511). Patient demographics and characteristics of BCxs collected, stratified by true positives, contaminants, and negative BCxs, are summarized in Table 1.

**Table 1.** Patient demographics of the hematologic malignancy population with blood cultures collected

Variable	True positive blood cultures (n = 111)	Negative blood cultures (n = 1,373)	Contaminants (n = 27)	Total blood cultures (*n* = 1511)
Age, median (range)	64 (23–86)	60 (20–91)	56 (24–84)	61 (20–91)
Male gender, n (%)	58 (52.3)	828 (60.3)	12 (44.4)	898 (59.4)
*Race, n (%)*				
Asian	4 (3.6)	14 (1)	0	18 (1.2)
American Indian	0	41 (3)	3 (11.1)	44 (2.9)
Black	24 (21.6)	345 (25.1)	4 (14.8)	373 (24.7)
White	83 (74.8)	920 (67)	20 (74.1)	1,023 (67.7)
Unknown	0	36 (2.6)	0	36 (2.4)
Other	0	17 (1.2)	0	17 (1.1)
*Ethnicity, n (%)*				
Non-Hispanic	103 (92.8)	1,176 (85.7)	21 (77.8)	1,300 (86)
Hispanic	4 (3.6)	89 (6.5)	4 (14.8)	97 (6.4)
Not reported	4 (3.6)	108 (7.9)	2 (7.4)	114 (7.5)
*Underlying diagnosis, n (%)*				
Aplastic anemia	3 (0.2)	16 (1.1)	0	19 (1.3)
Acute lymphocytic leukemia	14 (0.9)	173 (11.4)	10 (0.7)	197 (13)
Acute myelogenous leukemia	50 (3.3)	459 (30.4)	9 (0.6)	518 (34.3)
Chronic granulomatous disease	0	18 (1.2)	0	18 (1.2)
Chronic lymphocytic leukemia	0	23 (1.5)	0	23 (1.5)
Chronic myeloid leukemia/chronic myelomonocytic leukemia	13 (0.9)	28 (1.9)	1 (0.1)	42 (2.8)
Hodgkin lymphoma	0	52 (3.4)	0	52 (3.4)
Myelodysplastic syndrome/myeloproliferative neoplasms	3 (0.2)	84 (5.6)	1 (0.1)	88 (5.8)
Multiple myeloma/plasma cell leukemia	14 (0.9)	246 (16.3)	2 (0.1)	262 (17.3)
Non-Hodgkin lymphoma	13 (0.9)	244 (16.1)	2 (0.1)	259 (17.1)
Other^a^	1 (0.1)	30 (2)	2 (0.1)	33 (2.2)
*Applied cellular therapy^b^*, *n (%)*				
Autologous HCT	7 (6.3)	185 (13.5)	3 (11.1)	195 (12.9)
Allogeneic HCT	39 (35.1)	323 (23.5)	8 (29.6)	370 (24.5)
BCMA-directed CAR T-cell	3 (2.7)	82 (5.6)	1 (3.7)	86 (5.7)
CD19-directed CAR T-cell	0	54 (3.9)	0	54 (3.6)
*Specimen source, n (%)*				
Peripheral blood culture	62 (4.1)	849 (56.2)	10 (0.7)	921 (61)
Central line blood culture	49 (3.2)	524 (34.7)	17 (1.1)	590 (39)
*Patient location, n (%)*				
Inpatient	96 (6.3)	1,248 (82.6)	24 (1.6)	1,368 (90.5)
Outpatient	15 (1)	125 (8.3)	3 (0.2)	143 (9.5)

a Other includes: amyloid (n = 2), acute promyelocytic leukemia (n = 10), chronic eosinophilic leukemia (n = 2), mantle cell lymphoma (n = 1), mixed phenotype acute leukemia (n = 6), Waldenstrom’s macroglobulinemia (n = 3), and multiple sclerosis (n = 7).

b Represents the cellular therapy applied directly preceding the submission of blood cultures (where relevant). For patients receiving more than one directed cellular therapy, only the most recently applied is recorded.

BCMA, b-cell maturation antigen; CAR, chimeric antigen receptor; HCT, hematopoietic cell transplant.

The primary indications for BCx collection included neutropenic fever (49.1%), non-neutropenic fever (15.4%), documentation of BSI clearance (8.2%), and chills or rigors (5.6%). BCx yield varied substantially by indication. The highest yield occurred in cultures obtained for chills or rigors (23.8%), followed by suspected skin and soft tissue or bone infection (13.5%) and intra-abdominal infection (10.7%).

Among the 27 BCxs identified as contaminants, 16 (59%) were collected for the clinical indication of febrile neutropenia.

**Figure 1.**
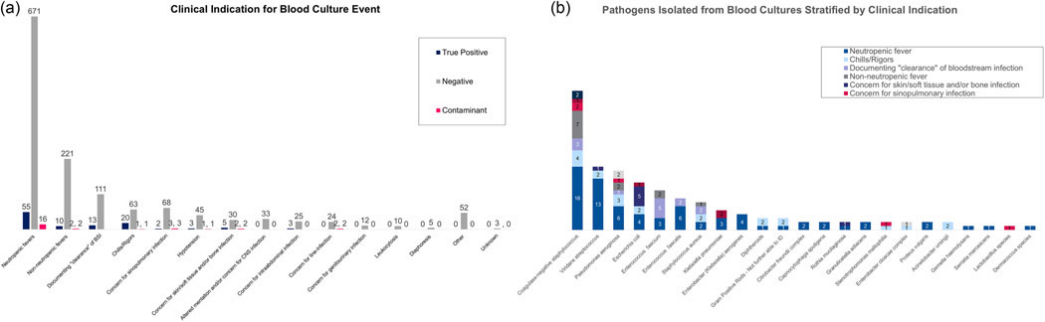
Figure 1 long description. BCx collection frequency and true-positive yield by clinical indication (a) and pathogens isolated from BCxs by clinical indication (b).

Coagulase-negative staphylococci (25.4%), viridans group streptococci (11.6%), and *Pseudomonas aeruginosa* (10.9%), were the most frequently isolated organisms, encompassing both true pathogens and contaminants. Indication-specific culture yields and organism distributions are shown in Figure 1, which combines clinical indication frequency with corresponding positivity rates.

## Discussion

This single-center study of patients with hematologic malignancies found that BCx yield varied by clinical indication, underscoring opportunities for diagnostic stewardship in a population frequently excluded from such algorithms. Although neutropenic fever accounted for nearly half of all BCx collection cases, the highest yield was observed among cultures obtained to evaluate patients with chills or rigors or to rule out dissemination of focal infections. These scenarios may warrant particular emphasis in stewardship frameworks for this patient population.

Most published BCx stewardship algorithms have been developed for and validated in general medical populations and explicitly exclude neutropenic and immunocompromised patients. The DISTRIBUTE study specifically excluded neutropenic patients from its intervention.^4^ Only recent studies begun to address this population.^5^ However, the algorithm used in our analysis was primarily adopted from the DISTRIBUTE study, that did not fully address the unique challenges faced in hematologic malignancy patients or patients with prolonged neutropenia and recurrent neutropenic fever.

Febrile neutropenia warrants specific attention. The largest study addressing this question included 620 patients with febrile neutropenia lasting >3 days and found that 25% had a positive BCx on day 1, whereas fewer than 5% per day had a new organism isolated on days 2–9.^6^ Among 503 patients with BCxs collected after day 3, only 19 (4%) had a new BSI detected. Of 31 organisms isolated after day 3, 26% were contaminants, further reducing diagnostic yield. Only 1 of 36 deaths within 30 days was attributable to a BSI detected after day 3, suggesting limited value of repeat serial BCxs in this patient population overall and in practice.

Allogeneic hematopoetic stem cell recipients demonstrated the highest BCx positivity rate in the cohort. This finding is biologically plausible given the prolonged immune dysfunction, graft-versus-host disease, immunosuppressive therapy, mucosal barrier injury, and central venous catheter exposure that characterize this population. Positive cultures occurred both early and late after transplantation, suggesting that the increased yield was not limited to the immediate posttransplant period.

The number of contaminant BCxs (27), particularly among patients with febrile neutropenia (16, 59%), is notable, as BCx contamination is associated with prolonged hospitalization, unnecessary antibiotic therapy, additional testing, increased costs, and patient harm.^7–9^ Moreover, data show a decline in BCx yield after the first 1–3 days of febrile neutropenia, with repeat cultures beyond day 3 offering little diagnostic benefit, suggesting that many of these contaminated cultures may have been avoidable.^6^

A novel aspect of this study is inclusion of outpatient BCxs. A Canadian study of 3,102 BCx sets from 1,732 ambulatory outpatients found a significant isolate yield of only 2.4.^10^ In our study, 143 (9.5%) BCxs were obtained outpatient; 125 (87.4%) were negative and 3 (2.1%) were contaminants, highlighting an opportunity for outpatient diagnostic stewardship interventions.

Periodic BCx audits not only inform diagnostic stewardship efforts, but also identify local epidemiologic trends, guide empiric antibiotic selection, and inform preventive strategies in patients with hematologic malignancies. Integrating surveillance into stewardship programs allows practices to evolve based on institution-specific real-world data rather than relying on extrapolation from generalized populations.

This study has several limitations. The retrospective design limits causal inference, and data collected over a four-month period at a single academic center may limit generalizability. Additionally, adjudication of BCx results may be subject to misclassification despite the use of standardized criteria. We had too much missing data to reliably adjudicate neutropenia on the day of BCx collection. Our analysis is based on BCx, not individual patients. Finally, patients with BCxs collected outside standard hematologic malignancy inpatient and outpatient settings were not included.

Despite these limitations, the findings inform BCx stewardship tailored to patients with hematologic malignancies. Scenario-specific approaches may reduce unnecessary testing while preserving timely identification of true BSIs in this vulnerable population. Future studies may provide a tailored algorithm for this patient population for the needs that current algorithms do not address.

## Figure 1. Long description

Panel A: A vertical bar graph shows the frequency and true-positive yield of blood culture collections by clinical indication. The x-axis lists various clinical indications, such as neutropenic fever, non-neutropenic fever, and others. The y-axis represents the number of blood culture events. The bars are color-coded to indicate true positive, negative, and contaminant results. The highest frequency is observed for neutropenic fever, followed by non-neutropenic fever and documented infection. Panel B: Another vertical bar graph displays the pathogens isolated from blood cultures stratified by clinical indication. The x-axis lists different pathogens, and the y-axis represents the number of isolates. The bars are color-coded to indicate different clinical indications. The most common pathogens are coagulase-negative staphylococci and viridans streptococci, with varying frequencies across different clinical indications.

Navigate back to Figure 1..

## References

[1] Attman E , Aittoniemi J , Sinisalo M , et al. Etiology, clinical course and outcome of healthcare-associated bloodstream infections in patients with hematological malignancies: a retrospective study of 350 patients in a Finnish tertiary care hospital. Leuk Lymphoma 2015;56:3370–3377. doi: 10.3109/10428194.2015.1032967. 25813080

[2] Chen S , Lin K , Li Q , et al. A practical update on the epidemiology and risk factors for the emergence and mortality of bloodstream infections from real-world data of 3014 hematological malignancy patients receiving chemotherapy. J Cancer 2021;12:5494–5505. doi: 10.7150/jca.50802. 34405012 PMC8364636

[3] Kimura SI , Gomyo A , Hayakawa J , et al. Clinical significance of repeat blood cultures during febrile neutropenia in adult acute myeloid leukaemia patients undergoing intensive chemotherapy. Infect Dis (Lond) 2017;49:748–757. doi: 10.1080/23744235.2017.1340665. 28631944

[4] Fabre V , Klein E , Salinas AB , et al. A diagnostic stewardship intervention to improve blood culture use among adult nonneutropenic inpatients: the DISTRIBUTE study. J Clin Microbiol 2020;58. doi: 10.1128/jcm.01053-20. PMC7512168 32759354

[5] Mera MIF , Oswalt C , McManus HD , Seidelman JL. Reducing unnecessary blood cultures in oncology through algorithmic stewardship in a tertiary medical center. Support Care Cancer 2025;33:780. doi: 10.1007/s00520-025-09835-6. 40790362

[6] Rosen EA , Krantz EM , Thibodeau A , et al. Diagnostic yield of repeat blood cultures and risk factors for bloodstream infection in persistent febrile neutropenia. Clin Infect Dis 2026:ciag014. doi: 10.1093/cid/ciag014. 41556483 PMC13536489

[7] Dempsey C , Skoglund E , Muldrew KL , Garey KW. Economic health care costs of blood culture contamination: a systematic review. Am J Infect Control 2019;47:963–967. doi: 10.1016/j.ajic.2018.12.020. 30795840

[8] Klucher JM , Davis K , Lakkad M , Painter JT , Dare RK. Risk factors and clinical outcomes associated with blood culture contamination. Infect Control Hosp Epidemiol 2022;43:291–297. doi: 10.1017/ice.2021.111. 33896442

[9] Liaquat S , Baccaglini L , Haynatzki G , Medcalf SJ , Rupp ME. Clinical consequences of contaminated blood cultures in adult hospitalized patients at an institution utilizing a rapid blood-culture identification system. Infect Control Hosp Epidemiol 2021;42:978–984. doi: 10.1017/ice.2020.1337. 33298207

[10] Laupland KB , Church DL , Gregson DB. Blood cultures in ambulatory outpatients. BMC Infect Dis 2005;5:35. doi: 10.1186/1471-2334-5-35. 15904503 PMC1156895

